# Supplementary material for: Black Ginseng Ameliorates Cellular Senescence via p53-p21/p16 Pathway in Aged Mice
Source: Biology (Basel). 2022 Jul 25;11(8):1108. doi: 10.3390/biology11081108 (PMC9331701; doi:10.3390/biology11081108)
Supplement: Supplementary file 1 [file biology-11-01108-s001.zip › biology-1619922-Table S1.pdf]

Table S1. Oligonucleotides for *real-time* RT-PCR.

Target gene	Gene ID	5' – 3'	Sequence
Cxcl2		Forward	5'-CCCAGACAGAAGTCATAGCCAC-3'
		Reverse	5'-TGGTTCTTCCGTTGAGGGAC-3'
IL-1β		Forward	5'-TGCCACCTTTTGACAGTGATG-3'
		Reverse	5'-TGATGTGCTGCTGCGAGATT-3'
IL-10		Forward	5'-CTTACTGACTGGCATGAGGATCA-3'
		Reverse	5'-GCAGCTCTAGGAGCATGTGG-3'
Mcp1		Forward	5'-GCATCCACGTGTTGGCTCA-3'
		Reverse	5'-CTCCAGCCTACTCATTGGGATCA-3'
Mmp12		Forward	5'-TGCACTCTGCTGAAAGGAGTCT-3'
		Reverse	5'-GTCATTGGAATTCTGTCCTTTCCA-3'
p16		Forward	5'-AACTCTTTCGGTCGTACCCC-3'
		Reverse	5'-GCGTGCTTGAGCTGAAGCTA-3'
p21		Forward	5'-CCTGGTGATGTCCGACCTG-3'
		Reverse	5'-CCATGAGCGCATCGCAATC-3'
Pai-1		Forward	5'-GACACCCTCAGCATGTTCATC-3'
		Reverse	5'-AGGGTTGCACTAAACATGTCAG-3'
Tnfaip2		Forward	5'-AGGAGGAGTCTGCGAAGAAGA-3'
		Reverse	5'-GGCAGTGGACCATCTAACTCG-3'
18s Ribosomal RNA		Forward	
		Reverse	

RT-PCR, reverse transcription polymerase chain reaction; Cxcl2, chemokine (C-X-C motif) ligand 2; IL, interleukin; Mcp1, monocyte chemoattractant protein 1; Mmp12, matrix metallopeptidase 12; Pai-1, plasminogen activator inhibitor-1; Tnfaip2, tumor necrosis factor alpha-induced protein 2.
