# Supplementary figures and images for: Black Ginseng Ameliorates Cellular Senescence via p53-p21/p16 Pathway in Aged Mice
Source: Biology (Basel). 2022 Jul 25;11(8):1108. doi: 10.3390/biology11081108 (PMC9331701; doi:10.3390/biology11081108)

D

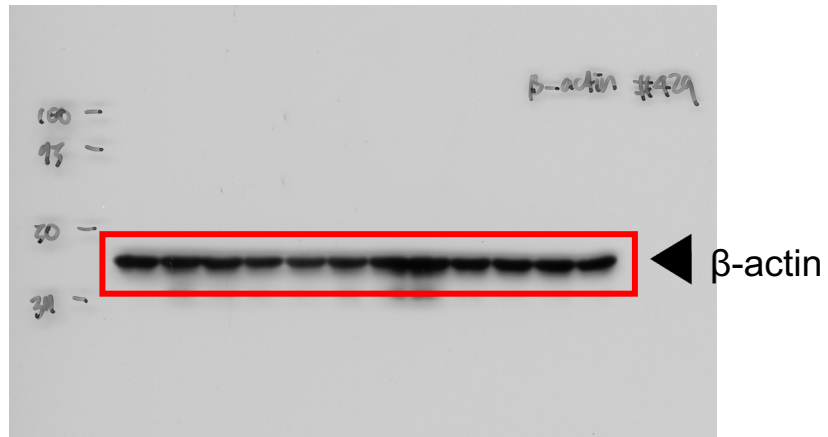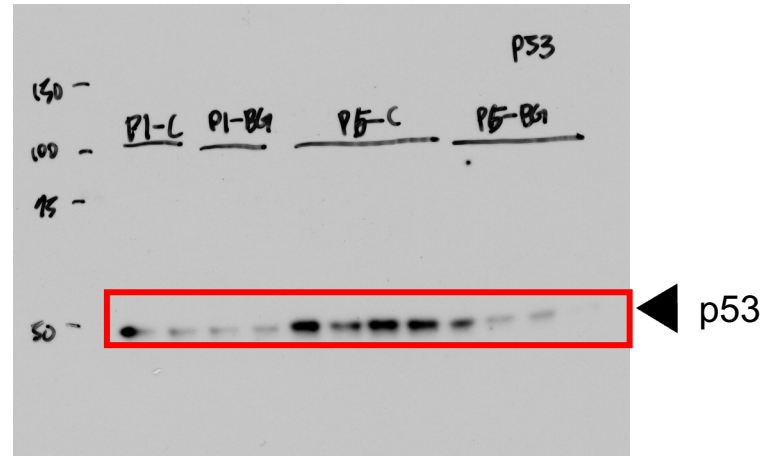

E

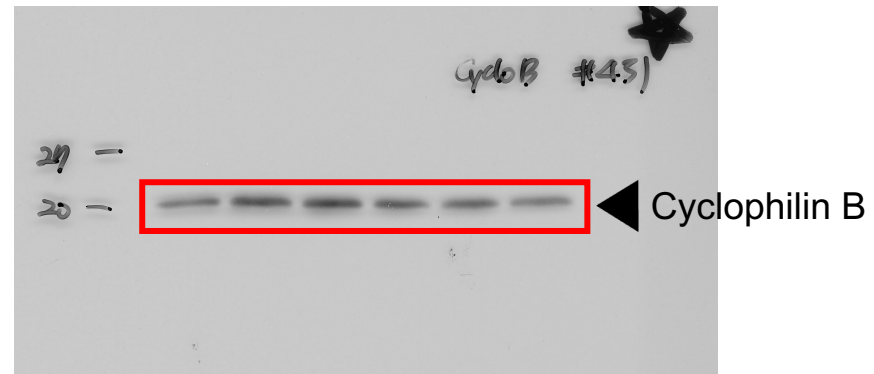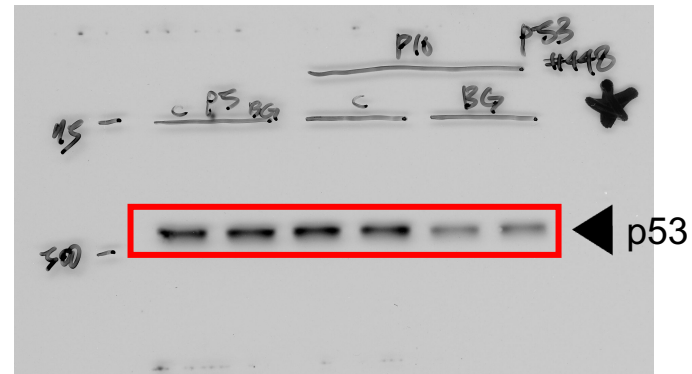

Figure 1D,E.

C

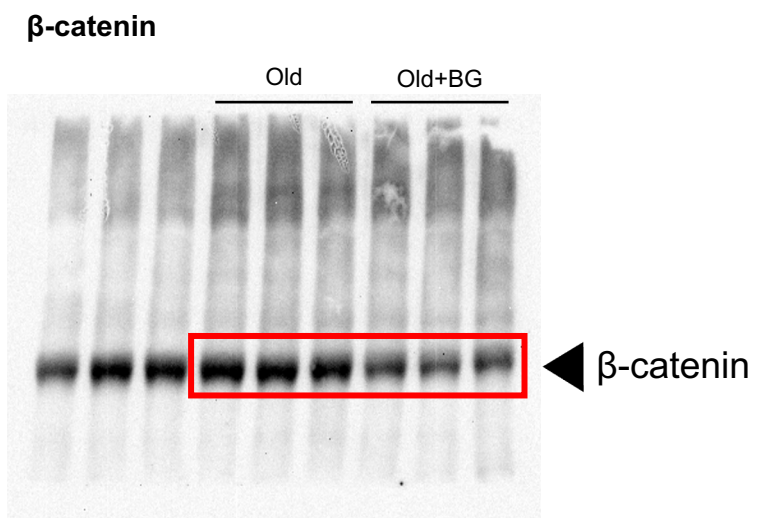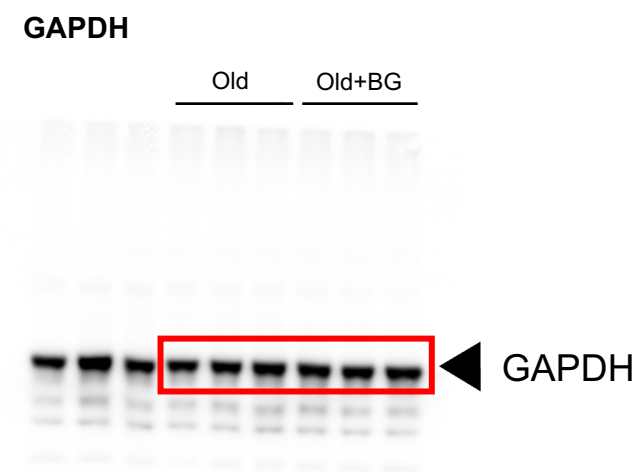

Figure 3C

A

p53

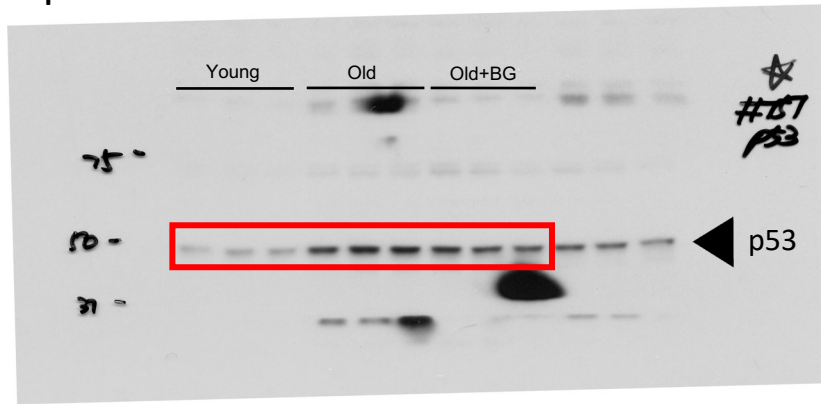

p21

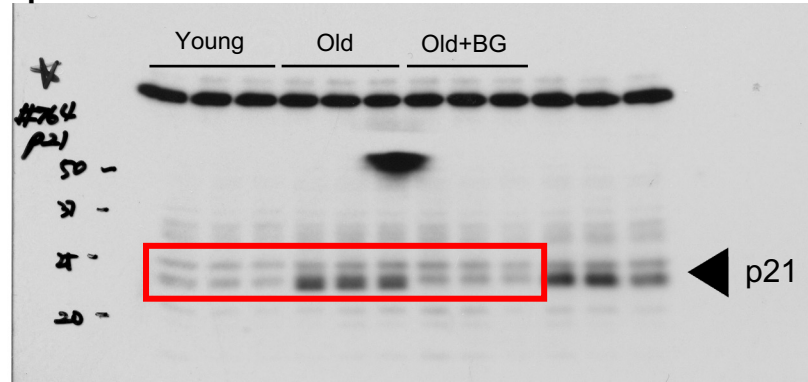

p16

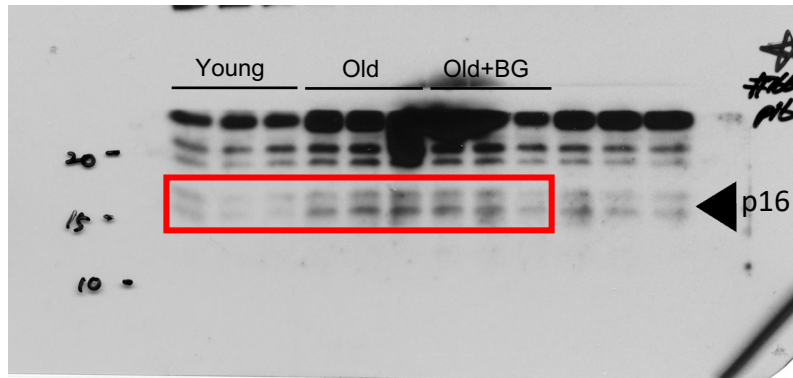

$\beta$ -actin

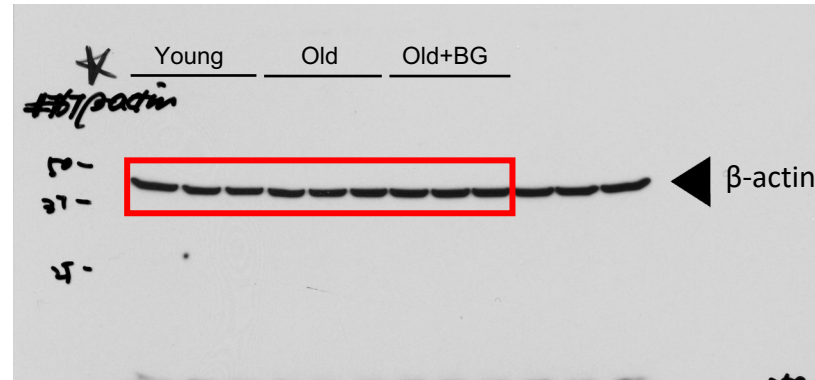

Figure 4A

B

p53

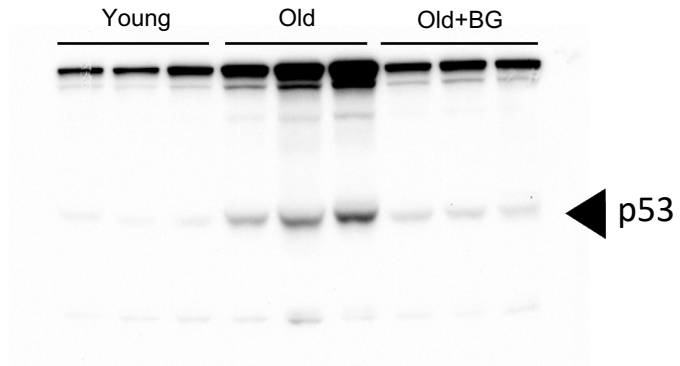

p21

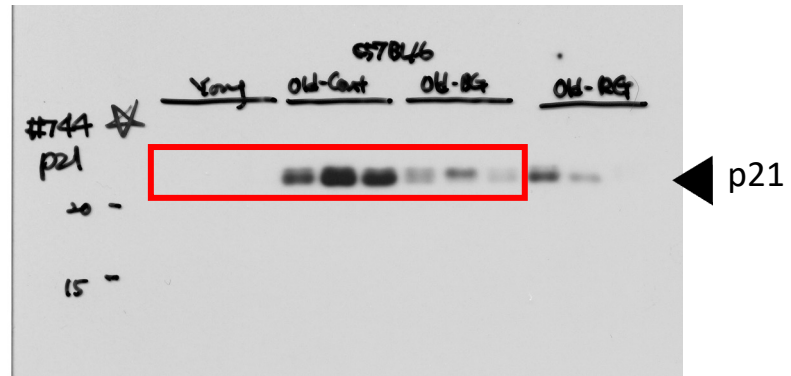

p16

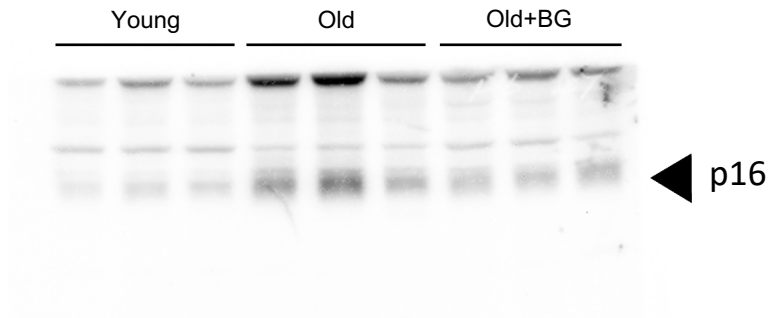

Cyclophilin B

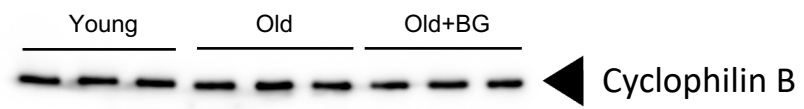

Figure 4B.

C

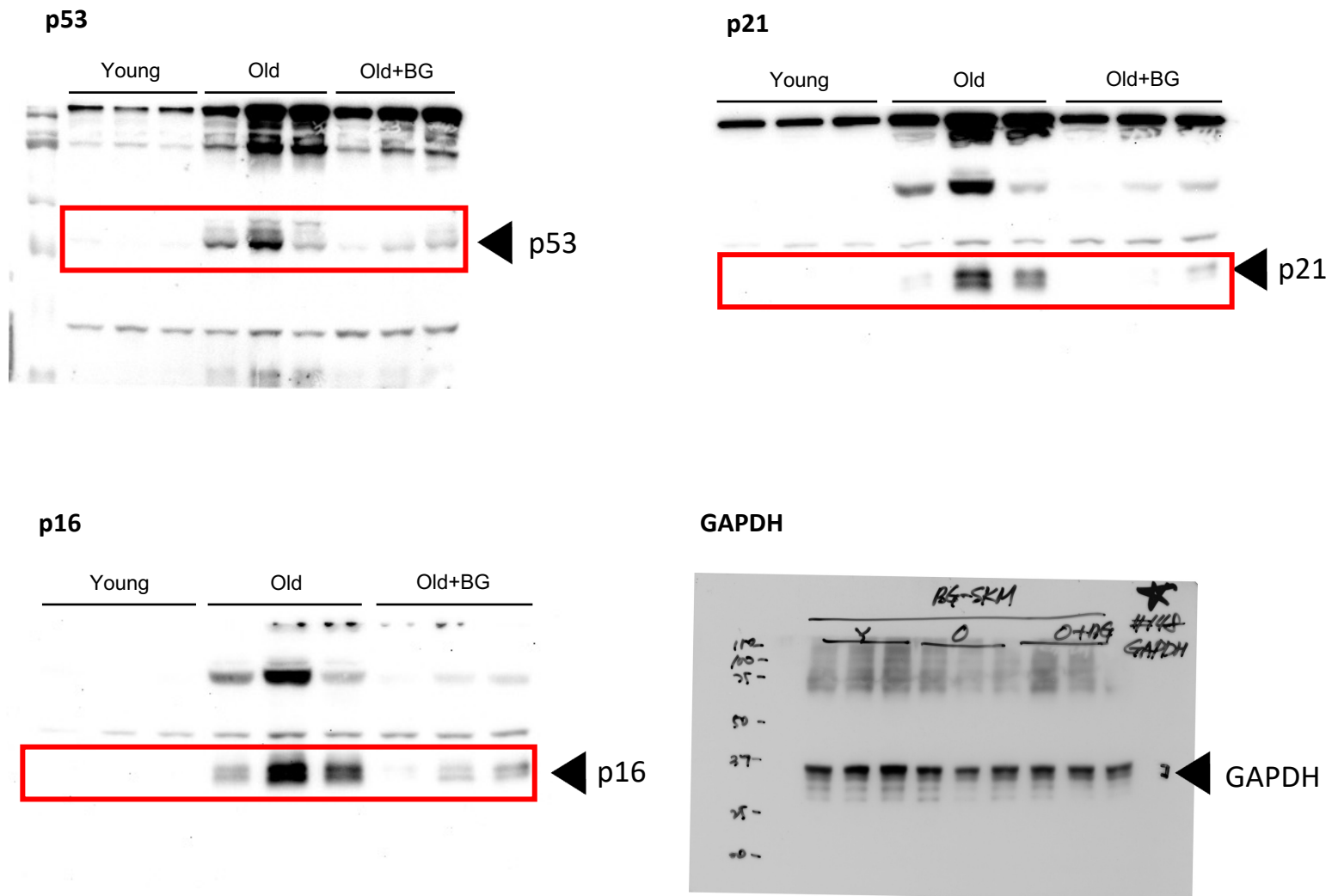

Figure 4C.

D

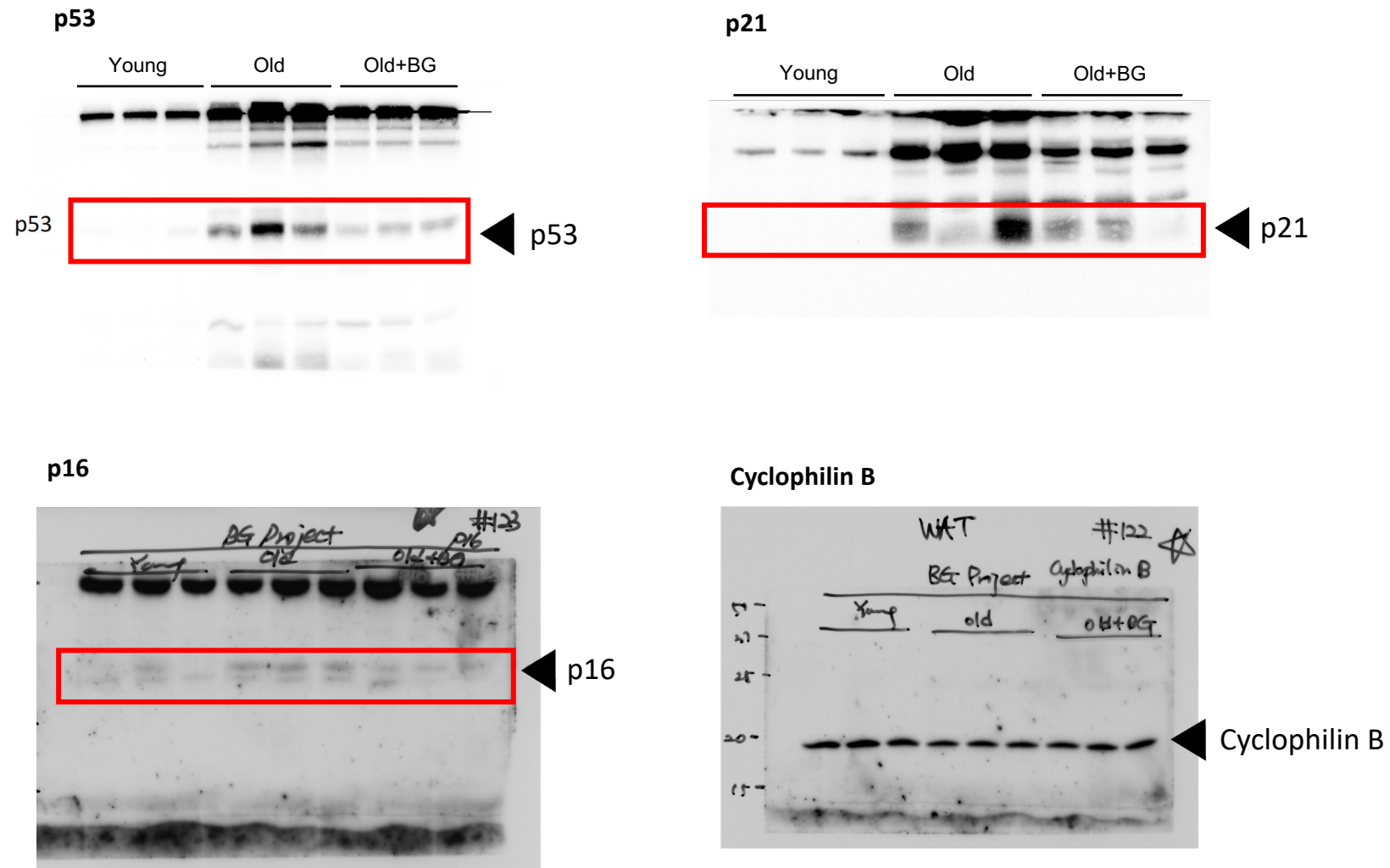

Figure 4D.

Supplement: Supplementary file 1 [file biology-11-01108-s001.zip › biology-1619922-File S1.pdf]
